# Features of affective disorders of the depressive spectrum after COVID-19 in patients with a history of mental disturbances

**DOI:** 10.1192/j.eurpsy.2025.1273

**Published:** 2025-08-26

**Authors:** N. O. Maruta, V. Y. Fedchenko, T. V. Panko, I. O. Yavdak, O. E. Semikina

**Affiliations:** 1 Borderline Psychiatry, “Institute of Neurology, Psychiatry and Narcology named after P.V. Voloshyn of NAMS of Ukraine” SI, Kharkiv, Ukraine

## Abstract

**Introduction:**

The structure of clinical manifestations in patients with a history of mental disturbances who suffered from COVID-19 and were exposed to the stressors of the SARS-CoV-2 pandemic is characterized by the predominance of affective disorders of the anxiety-depressive spectrum.

**Objectives:**

To investigate the structure of affective disorders of the depressive spectrum after COVID-19 in patients with a history of mental disturbances by nosological groups.

**Methods:**

95 patients with a history of mental disorders who have experienced COVID-19 were examined and made up the main group (F 32.0-32.2, 33.1, 33.2 – 31 patients, F 06.3, 06.4 – 33 patients, F 41.1, 41.2, 42.2, 45.3, 48.0 – 31 patients). During the study, a thorough analysis of the anamnesis of the disease was carried out, including information about the experienced coronavirus disease COVID-19. Clinical-psychopathological, clinical-amnestic, psychometric (the Montgomery-Asberg Depression Rating Scale (MADRS)) and methods of statistical analysis were applied.

**Results:**

According to the results of the psychodiagnostic study, the features of affective disorders of the depressive spectrum of various degrees of severity were established, which had certain differences according to nosological groups. Patients with depressive disorders have depressive disturbances in the form of sleep impairment (4.48 points, p = 0.0001), manifestations of sadness (4.27 points, p ≤ 0.021), internal tension (3.95 points, p = 0.01), difficulties in experiencing feelings (3.87 points, p ≤ 0.019), concentration difficulties (3.85 points, p = 0.031), appetite disturbances (3.11 points, p ≤ 0.012), manifestations of fatigue (3.06 points, p = 0.027), pessimistic (2.78 points, p ≤ 0.026) and suicidal thoughts (1.67 points, p = 0.038). Patients with mental disorders of organic genesis are characterized by depressive disturbances in the form of a feeling of fatigue (4.18 points, p ≤ 0.021), significant concentration difficulties (4.16 points, p = 0.024), sleep deterioration (3.87 points, p = 0.018), feeling and expressing sadness (3.16 points and 3.49 points, respectively), difficulties in experiencing feelings (2.43 points, p = 0.035). Patients with neurotic, stress-related and somatoform disorders are characterized by depressive manifestations in the form of expression of sadness (4.78 points, p = 0.034), feeling of internal tension (3.67 points, p = 0.027), concentration difficulties ((2 .68 ± 0.29) points).

**Conclusions:**

The results of the study of the structure of affective disorders of the depressive spectrum after COVID-19 in patients with a history of mental disturbances should be used as targets for effective therapeutic influence.

**Disclosure of Interest:**

None Declared

